# Strategy for Monitoring Cardiac Interventions with an Intelligent Robotic Ultrasound Device †

**DOI:** 10.3390/mi9020065

**Published:** 2018-02-02

**Authors:** Shuangyi Wang, James Housden, Areeb Zar, Ruchi Gandecha, Davinder Singh, Kawal Rhode

**Affiliations:** 1 School of Biomedical Engineering and Imaging Sciences, King’s College London, 4th Floor North Wing, St Thomas’ Hospital, London SE1 7EH, UK; richard.housden@kcl.ac.uk (J.H.); areeb.zar@kcl.ac.uk (A.Z.); ruchi.gandecha@kcl.ac.uk (R.G.); kawal.rhode@kcl.ac.uk (K.R.); 2 Xtronics Ltd., Gravesend, Kent DA12 2AD, UK; xtronics@me.com

**Keywords:** ultrasound robot, cardiac interventions, modeling and simulation, automatic control

## Abstract

In recent years, 3D trans-oesophageal echocardiography (TOE) has become widely used for monitoring cardiac interventions. The control of the TOE probe during the procedure is a manual task which is tedious and harmful for the operator when exposed to radiation. To improve this technique, an add-on robotic system has been developed for holding and manipulating a commercial TOE probe. This paper focuses on the probe adjustment strategy in order to accurately monitor the moving intra-operative catheters. The positioning strategy is divided into an initialization step based on a pre-planning method, and a localized adjustment step based on the robotic differential kinematics. A series of experiments was performed to evaluate the initialization and the localized adjustment steps. The results indicate a mean error less than 10 mm from the phantom experiments for the initialization step, and a median error less than 1.5 mm from the computer-based simulation experiments for the localized adjustment step. Compared to the much bigger image volume, it is concluded that the proposed methods are feasible for this application. Future work will focus on evaluating the method in a more realistic TOE scanning scenario.

## 1. Introduction

Catheter-based procedures for minimally invasive surgeries have been widely used in the last two decades as a replacement for open surgery for treating various types of heart disease. In these procedures, miniatured catheters (long, thin, flexible, hollow tubes), serving a broad range of functions for different treatment purposes, are slowly moved into the heart. The catheter is inserted into a large vein through a small incision, made usually in the groin area, and then is advanced into the heart. To guide the catheter’s operation within the heart, X-ray fluoroscopic imaging and 2D trans-oesophageal echocardiography (TOE) are typically the modalities of choice. Working collaboratively, fluoroscopy provides high-contrast images of the devices, while 2D TOE provides more detailed information on the soft tissues. With the development in recent years of real-time 3D ultrasound using matrix-array techniques, guiding of catheter-based procedures can be performed using a 3D TOE probe [1], and this approach has been gradually investigated by more organizations ever since the first experience reported in [2]. Though 2D TOE still gains wider acceptance among cardiologists and anesthetists, as it has been performed for many years, and has been carefully standardized for each step of different procedures [3], evidence has shown that 3D TOE has advantages in visualizing intra-operative catheters and heart structures with its volumetric imaging nature [4,5]. With the use of 3D TOE, the entire scenario, including the catheter, devices, and heart structures (e.g., septum, valve, and appendage) in most catheter-based procedures can be imaged in a single 3D view [6,7]. This feature enables 3D TOE potentially to be used more widely in the future for guiding cardiac interventional procedures, and standardized 3D protocols for specific procedures have already been investigated by researchers, such as described in [8].

Despite the advancement of the ultrasound imaging techniques, 3D TOE still remains as an operator-based manual approach. With the operator standing next to the X-ray system and manipulating the TOE probe, it is tedious and harmful for the duration of the longer interventional procedures [9]. Heavy protection clothes and long periods of standing are required, which can lead to several occupational diseases [10,11]. Additionally, the single 3D view for TOE still has a narrow field-of-view (FOV), and might require continuous adjustment to keep the catheter in a good position. To address these challenges, an add-on robotic system [12], which allows remote control of a commercial TOE probe (x7-2t, Philips, Amsterdam, The Netherlands) has been designed and manufactured. The robot has four degrees-of-freedom (DOFs), including the translation and rotation of the probe shaft, as well as the bi-directional bending of the probe head. The device was designed such that it does not require any change to the original TOE probe, which has the benefit of being able to use existing probes already employed clinically. With the probe initially positioned using all DOFs, it is expected the remaining minor adjustments could be achieved by the two DOF bi-directional bending. This configuration would be ideal for the automatic use of the device as the manipulation of the shaft of the probe, e.g., translation further down from the oral cavity, usually requires human assistance, while the bi-directional bending of the probe head, which happens in a local area, could be achieved automatically and remotely during the procedure.

In this paper, a new concept using the designed TOE robot to automatically monitor intra-operative catheters and place the catheter in the center of the FOV has been presented. To our knowledge, the device we designed was the first published system for robotically manipulating a TOE probe, and the automatic catheter monitoring with TOE is a unique topic which has not been reported before. Detailed methods employed in this paper are related to research work for other types of endoscopes in different applications, such as described in [13,14]. In addition, a probe steering method for an intra-cardiac ultrasound robotic system based on the inverse kinematics was proposed in [15], to automatically track targeted structures or devices with the catheter pointing towards the target accordingly. The automatic tracking was achieved by computing the angle between the target and the imaging plane and commanding a specific movement. In our work, the automatic adjustments of the TOE probe are done by a pre-planning method for initialization and a numerical servo loop based on differential kinematics for localized adjustment. This paper, extended from a previous published conference paper [16], is improved by including phantom experiments for the initialization step and using specially-defined clinical procedures, rather than randomly generated points, for the simulation study of the localized adjustment step.

## 2. Materials and Methods

### 2.1. Overview of the Robotic Trans-Oesophageal Echocardiography (TOE) System

**Figure 1:**
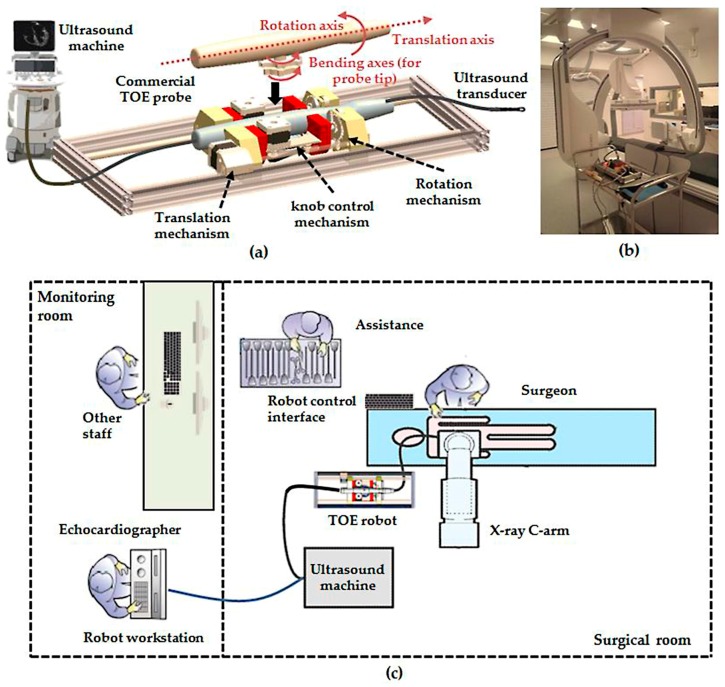
(**a**) Schematic drawing of the proposed add-on robotic trans-oesophageal echocardiography (TOE) system; (**b**) Photo of the implemented system in the surgical room with a trolley; (**c**) Floor plan of the potential use of the robotic TOE system in the surgical and monitoring rooms for cardiac procedures.

The developed add-on TOE robot (Figure 1a) holds the probe handle and manipulates four mechanical DOFs that are available in manual handling of a commercial probe. This includes translation of the probe, rotation of the probe, and bi-directional bending of the probe head. The additional DOF for electronic steering of the ultrasound beam, controlled by a pair of buttons on the side of the probe handle, is not robotized, as this function is also accessible from the ultrasound scanner for the proposed robotic use. The robot includes a handle control structure to rotate the two co-axial knobs with belt mechanisms built into an actuating chamber, a probe handle rotation structure to rotate the shaft about the long axis with a gear train mechanism actuated by a pair of motors, and a linear belt mechanism mounted on a rail to translate the probe along the long axis. With these mechanisms, the probe tip where the ultrasound transducer is located can be translated, rotated, and bent bi-directionally according to a set of robotic parameters. As human assistance is still important to insert the probe into the oral cavity and guide it down to the oesophagus, the translational axis was designed to allow both robotic and manual controls.

With microcontrollers and Bluetooth modules built inside the handle actuating chamber, the TOE robot can be remotely controlled by the user via Bluetooth communication from a PC. More details of the design, along with the method of operation and safety concerns, can be found in our previous work [12]. The whole system is designed to work as an individual piece next to the surgical bed, such as standing on a trolley (Figure 1b). Different options to manipulate the TOE robot have also been provided, including control from software, a gamepad, a dummy probe, and a haptic device. Particularly, the design and the use of a dummy probe are described in the work [17].

For the use of the proposed TOE robot, an exemplary embodiment is shown in Figure 1c with a remote-controlled actuation of a TOE probe and clinicians for the procedure. In this configuration, the TOE probe, manipulated by the proposed robot, is controlled by the echocardiographer (either a cardiologist or an anesthetist) from a separate room (e.g., the monitoring room) with a robot workstation. The workstation is structurally configured with hardware and software to generate motor commands to the TOE robot via different user inputs as introduced. The ultrasound images shown on the ultrasound machine inside the surgical room are also fed back to the robot workstation for the echocardiographer. Additionally, in a collaborative control scheme between the echocardiographer and the surgeon, appropriate user interfaces for facilitating additional control of the TOE probe from the surgeon could also be included.

### 2.2. Pre-Planning Method for Initialization

**Figure 2:**
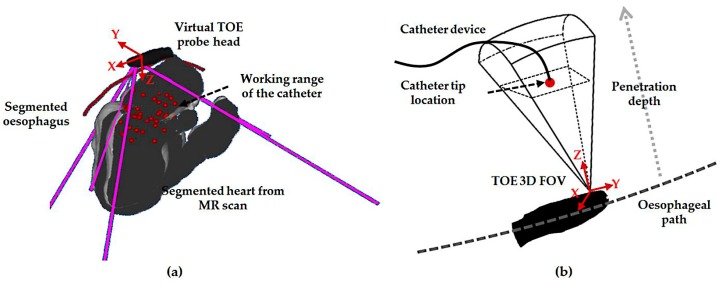
(**a**) Illustration of the initialization step using the view-planning platform to automatically decide the probe location in the approximately correct region; (**b**) Illustration of the localized probe adjustment step in order to continuously monitor the device in the center of the field-of-view (FOV).

The pre-planning method used for the initialization step intends to locate the global probe position during a defined procedure. In a view-planning platform developed for 3D TOE (Figure 2a), an automatically segmented 3D heart model from a pre-scanned magnetic resonance (MR) image, a manually segmented oesophagus center line, and the scanned model of the TOE probe head can be loaded and simulated. With the kinematics of the probe modelled, the corresponding virtual 2D center slice and the cone-sized 3D volume FOV are displayed [17]. In the platform, markers representing the location of the catheter that is to be monitored are defined from the pre-scanned MR image and used to determine the necessary monitoring range of a TOE view. Based on the middle of the defined markers for a procedure (denoted as the target), the desired robotic parameters are determined. Ideally, as a way of optimizing the target position in the FOV, the defined target should be positioned at the center of the TOE FOV. However, the Z-distance between the probe’s transducer face and the target, measured along the Z-axis of the ultrasound image coordinates, is constrained by the heart-to-oesophagus distance, and therefore, the Z-distance optimization is limited. Moreover, most of the structures of clinical interest, where devices are likely to be placed, are already at good positions in the ultrasound image space (near field). Therefore, the focus of this work is on optimizing the X-axis distance and Y-axis distance measured along the X- and Y-axis of the ultrasound image coordinates, in which case the two distances are zero if the target is placed at the center of the X–Y plane.

Using gradient-descent search, an objective function based on the X- and Y-axial distances from the origin of the ultrasound image coordinates to the target can be defined to optimize the robotic parameters based on the forward kinematics [12]. The forward kinematic model *F*(**p**), where **p** is the robotic parameter set, gives the transformation between the patient coordinates and the predicted probe tip coordinates:

(1) $${}^{Patient}T_{ProbeTip}(p)=F(p)$$

The patient coordinates are defined from the MR image coordinates. The position vector of the target defined in the patient coordinates is denoted as **^Patient^t_Target_**. The position of the target in the ultrasound image coordinates can be calculated using a known calibration obtained from [18]:

(2) $${}^{US}t_{Target}(p)={}^{US}T_{ProbeTip}{}^{ProbeTip}T_{Patient}(p){}^{Patient}t_{Target}$$

Since both X- and Y-components of this position vector are expected to be zero, to locate the target at the center, the objective function is defined as the root sum square of the X- and Y-components obtained from **^US^t_Target_**(**p**). The search strategy would result in the best-fit probe robotic parameters p* to represent the desired probe pose ^Patient^T_ProbeTip_*. For the initialization, the process is performed by the operator using the robot, and the view will then be pointing to the approximate center of the catheter’s working range as the result of the initialization step.

### 2.3. Differential Kinematics for Localized Adjustment

**Figure 3:**
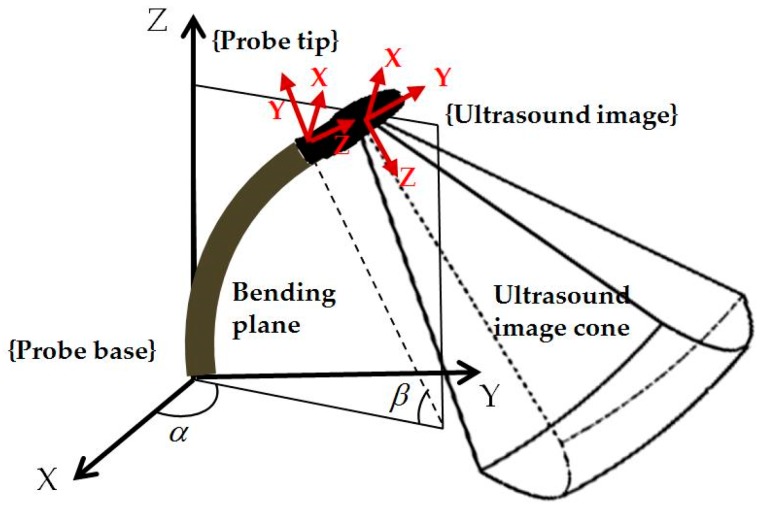
Geometric illustration of the TOE probe tip bending with the coordinates of the probe base, probe tip, and ultrasound image shown. The probe tip coordinate frame is defined on the rigid section of the probe head (based on a nano-CT scan of the probe) and the ultrasound image coordinate frame was defined by a fixed calibration from the probe tip coordinates.

For localized adjustment of the TOE probe (Figure 2b) to monitor the catheter after the initialization, differential kinematics and image servoing methods are utilized to adjust the bi-directional bending. This starts from investigating the forward kinematics of the bi-directional bending, which has been previously described in [12]. The inputs are the rotation angle of the first bending knob, *φ_x_*, which controls the bending tip pitch in the posterior–anterior plane; and the rotation angle of the second bending knob, *φ_y_*, which controls the bending tip yaw in the left–right plane. The geometric illustration of TOE probe tip bending is shown in Figure 3. The translation vector and rotation matrix between the probe tip coordinates and the probe base coordinates are expressed as

(3) $${}^{ProbeBase}t_{ProbeTip}=\left[\begin{array}{c} \frac{L_{f}}{\beta }(1-C_{\beta })C_{\alpha }+L_{u}S_{\beta }C_{\alpha }\\ \frac{L_{f}}{\beta }(1-C_{\beta })S_{\alpha }+L_{u}S_{\beta }S_{\alpha }\\ \frac{L_{f}}{\beta }S_{\beta }+L_{u}C_{\beta } \end{array}\right]$$

(4) $${}^{ProbeBase}R_{ProbeTip}=\left[\begin{array}{ccc} S^{2}{}_{\alpha }+C_{\beta }C^{2}{}_{\alpha } & -S_{\alpha }C_{\alpha }(1-C_{\beta }) & C_{\alpha }S_{\beta }\\ -S_{\alpha }C_{\alpha }(1-C_{\beta }) & C^{2}{}_{\alpha }+C_{\beta }S^{2}{}_{\alpha } & S_{\alpha }S_{\beta }\\ -C_{\alpha }S_{\beta } & -S_{\alpha }S_{\beta } & C_{\beta } \end{array}\right]$$

where *α* is the angle between the bending plane and the X–Z plane, *β* is the bending angle in the bending plane, *L_f_* is the length of the bending section of the probe tip, and *L_u_* is the length of the rigid section of the probe tip. In the matrix, *Sx* and *Cx*, respectively, denote sin(x) and cos(x).

For the differential kinematics, the robotic joint space parameters are defined as **R** = [*α*, *β*]*^T^*. The velocity skew of the probe tip **^ProbeTip^V** = [**v**, **ω**]*^T^*, expressed in the probe tip coordinates, is determined by the robotic Jacobian **J_R_**, where **^ProbeTip^V** = $J_{R}\overset{•}{R}$. The Jacobian of the position vector is expressed as

(5) $$J_{v}=\left[\begin{array}{cc} -\frac{L_{f}}{\beta }S_{\alpha }(1-C_{\beta })-L_{u}S_{\alpha }S_{\beta } & \frac{L_{f}}{\beta^{2}}C_{\alpha }(1-C_{\beta })+L_{u}C_{\alpha }\\ \frac{L_{f}}{\beta }C_{\alpha }(1-C_{\beta })+L_{u}C_{\alpha }S_{\beta } & \frac{L_{f}}{\beta^{2}}S_{\alpha }(1-C_{\beta })+L_{u}S_{\alpha }\\ 0 & -\frac{L_{f}}{\beta^{2}}S_{\beta }+\frac{L_{f}}{\beta } \end{array}\right]$$

The Jacobian for **ω** can be extracted from the skew-symmetry matrix, which is obtained from the multiplication of the partial derivative of **^ProbeBase^R_ProbeTip_** and the transpose of **^ProbeBase^R_ProbeTip_**:

(6) $$J_{w}=\left[\begin{array}{cc} -C_{\alpha }S_{\beta } & -S_{\alpha }\\ -S_{\alpha }S_{\beta } & C_{\alpha }\\ -1+C_{\beta } & 0 \end{array}\right]$$

The resulting robotic Jacobian is expressed as **J_R_** = [**J_v_**, **J_ω_**]*^T^*. The image servoing control for adjusting the probe bi-directional bending pointing to the catheter can be further determined by the image Jacobian, which relates differential changes in the image features to differential changes in the configuration of the transducer, i.e., the velocity skew **^ProbeTip^V**.

Let **^US^p** = [^US^*x,* ^US^*y,* ^US^*z*]*^T^* be the tracked catheter tip position in the 3D ultrasound image space. The coordinates of the catheter tip in the probe tip coordinates **^ProbeTip^p** are decided by the fixed calibration **^ProbeTip^p** = **^ProbeTip^T_US_^US^p**. In this study, the catheter tip position is assumed to be tracked either by image-based processing methods for **^US^p** or sensor-based tracking methods for both the TOE probe and the catheter, giving **^ProbeTip^p** as the result. The following relation describes the coordinates of the catheter tip in the probe base coordinate frame **^ProbeBase^p**:

(7) $${}^{ProbeBase}p={}^{ProbeBase}t_{ProbeTip}+{}^{ProbeBase}R_{ProbeTip}{}^{ProbeTip}p$$

**Figure 4:**
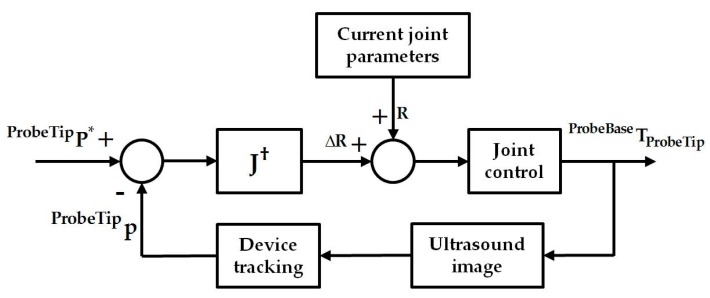
Jacobian position control loop for localized adjustment of the probe to monitor the catheter. In the diagram, **J^†^** is the pseudo-inverse of the final Jacobian **J**.

As the probe shaft is not expected to move during the localized adjustment, the catheter tip is considered not moving in the probe base coordinates for a given catheter tip position when bi-directional bending is applied (oesophagus movements are ignored). The rest of the components in (7) will all change when the bi-directional bending is applied. Taking the time derivative of (7), the following relationship is obtained according to the visual servoing method summarized in [19]:

(8) $$\overset{•}{{}^{ProbeBase}P}=\left[\begin{array}{cc} -I & S({}^{ProbeTip}p) \end{array}\right]{}^{ProbeTip}V=\left[\begin{array}{cc} -I & S({}^{ProbeTip}p) \end{array}\right]J_{R}\overset{•}{R}$$

where *S*(**^ProbeTip^p**) is the skew-symmetry matrix of **^ProbeTip^p**. The final Jacobian **J** relating the changes of robotic joint parameters to the changes of the catheter tip position in the probe tip coordinates is therefore obtained. With the differential kinematics, we have considered a simple controller as shown in Figure 4, where ^ProbeTip^P* is the ideal location of the catheter in the probe tip coordinates. This can be determined in the same way as described in the previous section so that the X- and Y-components for the ^US^P* are zero, while the Z-component remains at the same value for its current position in the ultrasound image coordinates. The corresponding desired position ^ProbeTip^P* can then be calculated based on the fixed calibration **^ProbeTip^T_US_** and used for the bi-directional position controller. A simple geometrical relationship then relates the resulting **R** = [*α*, *β*]*^T^* to the desired motor parameters for the bi-directional bending axes.

### 2.4. Simulation and Phantom Experiments for the Initialization Step

To verify the correct working of the initialization step, a computer-based simulation experiment focusing on the search strategy itself has been performed. Five hundred random points were defined within a heart–oesophagus segment obtained clinically to simulate the targets defined by the user. The probe positioning method described in Section 2.2 was applied to calculate the desired probe pose to image the defined target, which is the center of the catheter’s moving range, ideally placed at the center of the ultrasound FOV. Based on the resulting target’s position in the ultrasound image coordinates, the error representing the offset distance from the center of the ultrasound FOV can be quantified.

**Figure 5:**
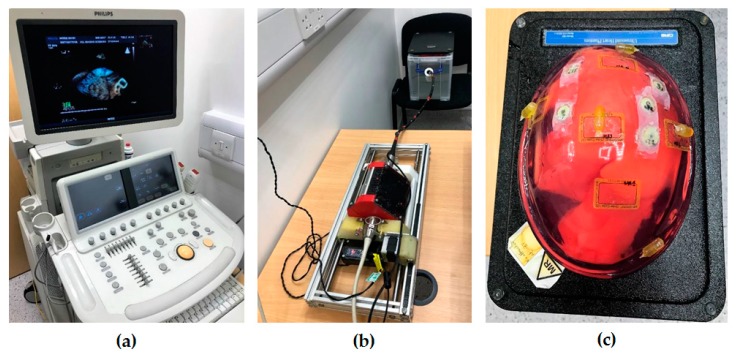
Experimental setup for the initialization step: (**a**) ultrasound machine; (**b**) TOE robot and the phantom setup; and (**c**) the heart phantom used for ultrasound imaging.

To simulate the initialization procedure in a more realistic setup as a proof of concept, an experiment using a custom-made heart–oesophagus phantom was performed (Figure 5). The phantom was MR scanned and imported to the view-planning platform. As the custom-made phantom has a different anatomical relationship towards the real heart and oesophagus, simulated procedures are manually defined based on the targeted anatomical structures, with six views selected for the experiment. For each view, the probe positioning method described in Section 2.2 was applied to calculate the desired probe tip pose to image the defined target. With the ideal view pre-planned, the probe positioning parameters were recorded. During the experiment, the probe was attached to the robot manually by the operator. Based on the depth scales marked on the original probe, the operator manually inserted the probe into the phantom cavity using the passive translation feature to the approximate distance as pre-planned. The active axes for rotation and bending of the probe driven by the robot were then utilized, by controlling from a PC based on the pre-planned angles and visual feedback and comparing the ultrasound images (center slices) to the pre-planned views. These 3D TOE views were acquired using the ultrasound machine (iE33 xMatrix, Philips, Amsterdam, The Netherlands), and the images were streamed to a PC via TCP/IP for post-processing.

### 2.5. Simulation Experiments for the Localized Adjustment Step

**Figure 6:**
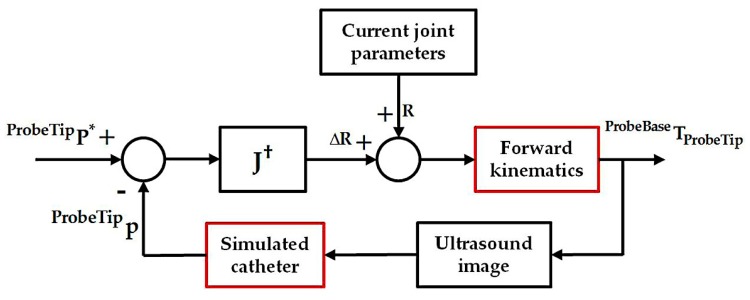
Overview of the simulation experiment using the Jacobian position control loop for localized adjustment with the red boxes highlighting the simulated sections.

To verify the correct working of the localized adjustment step, a computer-based simulation experiment was performed by simulating the catheter’s position vector **^US^p** = [^US^*x,* ^US^*y,* ^US^*z*]*^T^* in the ultrasound image space. The Z-component ^US^*z* was assigned within the depth of 5–10 cm, where most structures in a TOE scan are located. For the X- and Y-components indicating the in-plane movement range of the catheter, uniformly distributed simulated positions in the range from −5 cm to +5 cm were assigned to cover the size of a normal heart. Compared to Figure 4 described for the real scan, the experiment in this study uses the simulated catheter position as a way to replace the tracking of the catheter in a real scenario. For the joint control and bi-directional bending mechanism actuated by the robot in reality, this is replaced by the forward kinematics to generate the **^ProbeBase^p_ProbeTip_**. The experiment is summarized in Figure 6. Similar to the previous simulation, 500 random catheter positions were generated for the analysis. The corresponding resulting catheter tip positions in the ultrasound image coordinates, after applying the Jacobian position control loop, were recorded.

To further simulate the initialization step and the localized adjustment step in a more realistic clinical scenario, selected cardiac interventional procedures using 3D TOE as the monitoring device were simulated with catheter tip positions defined (608 points in total) based on the clinically obtained heart–oesophagus segment. Examples include atrial septal defect repair, left atrial ablation, percutaneous mitral valve repair or replacement, and trans-catheter aortic valve replacement (TAVR). The virtual catheter tip positions were defined in the original MR image of the patient based on the anatomies of the procedure. These points were then imported to the simulation platform to represent the targets to be monitored by the TOE image. Based on the simulated positioning data of the catheter tip, similar experiments as described for the randomly generated targets were re-performed, and the resulting positioning data were recorded. As the heart phantom used in Section 2.4 is a closed system which does not allow any medical instrument (such as catheters) to be placed inside, the phantom experiment for the localized adjustment step is currently not available.

## 3. Results and Discussion

### 3.1. Simulation and Phantom Experiments for the Initilization Step

**Figure 7:**
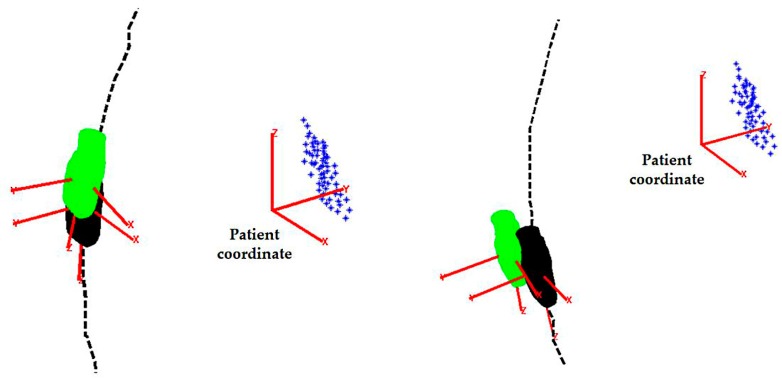
Examples of the pre-planned probe locations (probe head in black) and the real obtained probe locations (probe head in green) from the experiment with the pre-defined targets to be monitored (markers in blue) shown. The probe tip and the patient coordinates are shown in red.

For the simulated initialization method, the error function is defined as the root sum square of the X- and Y-components extracted from the matrix. The Z-component along the penetration depth of the ultrasound beam is not included as it usually has a minimum influence on the visualization. For the simulated experiments with randomly generated target position, the mean error was found to be less than 0.5 mm. To assess the performance of the acquisition for the phantom experiments, the acquired ultrasound images were registered to the pre-scanned MR images based on the method described in our previous work [17]. This calculates the real probe poses which can be used to derive the resulting target position in the ultrasound image coordinates. Therefore, the error representing the offset distance from the center of the ultrasound FOV can be quantified using the root sum square of the X- and Y-components. The mean error for the offset distance was found to be 8.36 ± 2.29 mm (mean ± standard deviation (SD)). Examples of the pre-planned and the real obtained probe locations are shown in Figure 7.

### 3.2. Simulation Experiments for the Localized Adjustment Step

For the localized adjustment method, the X- and Y-components extracted from the recorded catheter tip positions in the ultrasound image coordinates were compared with the desired values, in which case, both distances are expected to be zero. These are treated as the absolute errors. Since the pyramid FOV of 3D TOE extends with increasing ultrasound penetration depths, centralization of the catheter tip positioning in the X–Y planes at different penetration depths were analyzed, taking the size of the X–Y planes into consideration. This was done by calculating the ratio of the X- and Y-components to the half-length of the X–Y plane, measured along the X- and Y-axis of the live 3D TOE image space. These two ratios are defined as the normalized errors.

**Figure 8:**
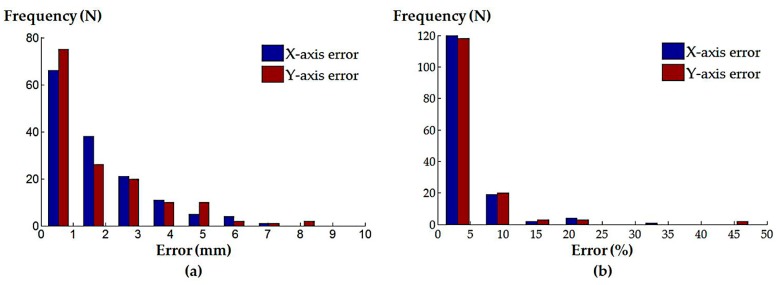
Histograms of the (**a**) absolute positioning errors and (**b**) normalized positioning errors for the randomly generated catheter tip positions.

**Figure 9:**
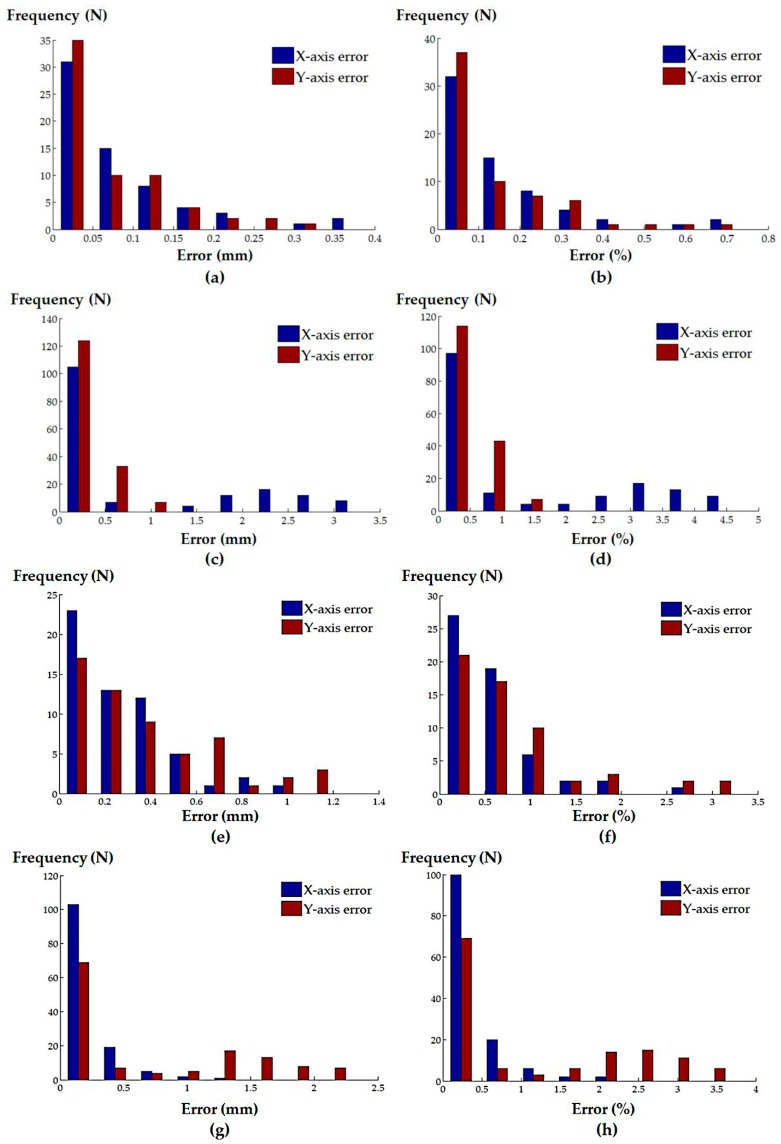
Histograms of the (**a**) absolute and (**b**) normalized positioning errors for the simulated atrial septal defect repair procedure; (**c**) absolute and (**d**) normalized positioning errors for the simulated left atrial ablation procedure; (**e**) absolute and (**f**) normalized positioning errors for the simulated mitral valve repair/replacement procedure; (**g**) absolute and (**h**) normalized positioning errors for the simulated trans-catheter aortic valve replacement procedure.

The median absolute and normalized errors of the X- and Y-components for both the randomly generated catheter positions and the defined clinical procedures were calculated, and are summarized in Table 1. The histograms of the absolute and normalized errors of the X- and Y-components for the randomly generated catheter locations are shown in Figure 8; corresponding histograms for the defined clinical procedures are shown in Figure 9.

For the above analyses, the results from the simulation of the initialization step indicate the correct working of the method on its own, with a submillimetre probe positioning error identified. Though the simulation experiments have verified the accurate working of the proposed initialization method, differences between the simulation environments and the real scanning scenarios, as well as the involvements of the operator, could potentially influence the performance of the method. This can be observed from the experimental results of the phantom experiment, where the pre-planning environment is different to the real scanning setup in terms of physical constraints. For this initialization step, though the pre-planning method can accurately find an ideal probe pose, guiding the probe to the exact location without real-time tracking of the TOE probe would be difficult. The issues for probe tracking have been discussed in detail in our previous work [17,20] where an image-based approach and a sensor-based tracking approach were investigated. In practice, the accuracy of the initialization is not crucial as it is an approximate process for large motions, while the localized adjustments are the main tools for accurate probe positioning. Therefore, no additional feedback is utilized in this work for the initialization step with the currently identified error level.

The results from the simulation of the localized adjustment process indicate that all absolute errors are less than 10 mm, and the majority of errors are less than 1 mm for both of the randomly generated positions and simulated clinical procedures. The errors are further interpreted by comparing to the size of the plane perpendicular to the ultrasound penetration direction in the live 3D TOE FOV. The normalized positioning errors indicate that all catheter tip positions are located in the center area of the FOV, when compared with the size of the live 3D volume. These results demonstrate the errors of the method on its own would have little influence on the operator’s visualization when observing the 3D image and the initial goal to automatically keep the catheter at the center of the FOV is achievable using the method, assuming the catheter can be tracked in the image coordinates. Comparing the results from the randomly generated positions to the simulated clinical procedures, lower errors have been identified for the simulated clinical procedures as the more clinically realistic approach in defining the catheter tip position resulted in a smaller required monitoring range of the TOE probe. A similar trend has been identified when comparing the three procedures within the clinical simulation experiment: a smaller monitoring range, e.g., the atrial septum repair procedure, results in smaller errors. This is because the probe’s bi-directional bending axes would have a higher possibility of success when used to cover a smaller required FOV. Therefore, the combined use of the initialization process and localized adjustment process is also important for accurate monitoring. Comparing Figure 4 with Figure 6, there are two main differences in the simulation experiment compared to the real scan. The real physical robotic movements for the bi-directional bending were replaced by the forward kinematic model to decide the probe pose. In reality, the performance of the bending axes is likely to be different to the prediction of the kinematics due to the complexities of the real environment. In addition, the catheter tip is assumed to be not moving in the probe base coordinates for a given catheter tip position when bi-directional bending is applied, while in reality, this may not be true and a time-dependent oesophagus movement might need to be included into the model.

The tracking of the catheter, either based on an image processing technique or an additional sensor, is assumed to be known in the simulation experiments. In reality, intra-operative instruments are difficult to visualize using ultrasound, due to image artefacts [21,22]. However, existing methods have suggested the possible approach of fast catheter tracking from 3D echo sequences based on segmentation from corresponding X-ray fluoroscopy and X-ray to ultrasound registration [23,24]. Additionally, a sensor-based approach using electromagnetic (EM) tracking can also be used when both the intra-operative device and the TOE probe are EM-tracked. Related works, such as described in [20,25], have discussed the integration of EM sensing with the TOE probe. The device navigation using EM tracking for interventions has been a hot topic, and different studies have been presented to demonstrate the approach, such as described in [26]. These works could be eventually integrated into the currently presented robotic methods to formulate a complete solution for the use of the TOE robot to monitor intra-operative devices.

It should also be noted that the initialization method and the simulated TOE procedures described in this study focused on trans-oesophageal views, such as upper-oesophageal views and mid-oesophageal views. The remaining trans-gastric views and deep trans-gastric views were not included, as these views were outside the segmented region of the current heart–oesophagus model obtained from the clinical image, as well as not available from the custom-made heart–oesophagus phantom. However, the current method can be adapted to work with the trans-gastric views without too much additional work. For the initialization step, to decide a global location of the probe, the current pre-planning strategy within the oesophagus using the gradient descent search method will be extended to the stomach. In the new scheme, the constraint of the probe’s location will not be limited to the oesophagus center line, but extended to the stomach with a virtual moving trajectory line of the probe generated from the geometries of the oesophagus and the stomach. For the localized adjustments using the proposed differential kinematics, the method will remain the same as the modelled localized space, since the derivation of the differential kinematics has no difference between trans-oesophageal and trans-gastric views.

## 4. Conclusions

This paper has described a strategy for controlling a recently developed TOE robotic system to adjust the pose of the probe, and therefore, automatically monitor intra-operative catheters used in cardiac interventions. The method has been divided into two stages with an approximate pose of the probe determined using a pre-planning method and an accurate pose of the probe adjusted based on a Jacobian position control loop. Accordingly, all of the four DOFs are employed for the once-only initialization, when human assistance is required for the probe insertion, and only two bending DOFs are utilized for the localized adjustments, when the robot could be controlled automatically. A series of experiments were performed, and the proposed method has been validated for its feasibility for use in this application. Limited by the experimental setup to simulate the complete workflow, our future works will focus on developing a new ultrasound imaging phantom to simulate the real trans-oesophageal approach, allowing both the TOE probe and a catheter to work inside the phantom in order to further test the proposed method.

## Figures and Tables

**Table 1:** Summary of the simulation experiment results for the localized adjustment step with the catheter tip positions defined from randomly generated positions and simulated clinical procedures.

Simulated Procedures	Axes	Absolute Error (Median ± InterQuartile Range (IQR))	Normalized Error (Median ± IQR)
Randomly generated catheter locations	X-axis	1.16 ± 2.09 mm	2.78 ± 4.01%
	Y-axis	1.03 ± 2.42 mm	2.18 ± 4.60%
Atrial septal defect repair	X-axis	0.05 ± 0.09 mm	0.09 ± 0.16%
	Y-axis	0.05 ± 0.10 mm	0.08 ± 0.17%
Left atrial ablation	X-axis	0.16 ± 1.87 mm	0.29 ± 2.69%
	Y-axis	0.14 ± 0.38 mm	0.26 ± 0.61%
Mitral valve repair/replacement	X-axis	0.20 ± 0.30 mm	0.43 ± 0.54%
	Y-axis	0.28 ± 0.40 mm	0.53 ± 0.85%
Trans-catheter aortic valve replacement	X-axis	0.09 ± 0.22 mm	0.15 ± 0.38%
	Y-axis	0.22 ± 1.37 mm	0.41 ± 2.23%

## References

[1] Vegas A., Meineri M. (2010). Core review: Three-dimensional transesophageal echocardiography is a major advance for intraoperative clinical management of patients undergoing cardiac surgery: A core review. Anesth. Analg..

[2] Perk G., Lang R.M., Garcia-Fernandez M.A., Lodato J., Sugeng L., Lopez J., Knight B.P., Messika-Zeitoun D., Shah S., Slater J. (2009). Use of real time three-dimensional transesophageal echocardiography in intracardiac catheter based interventions. J. Am. Soc. Echocardiogr..

[3] Hahn R.T., Abraham T., Adams M.S., Bruce C.J., Glas K.E., Lang R.M., Reeves S.T., Shanewise J.S., Siu S.C., Stewart W. (2013). Guidelines for performing a comprehensive transesophageal echocardiographic examination: Recommendations from the American society of echocardiography and the society of cardiovascular anesthesiologists. J. Am. Soc. Echocardiogr..

[4] Faletra F.F., Ho S.Y., Auricchio A. (2010). Anatomy of right atrial structures by real-time 3d transesophageal echocardiography. JACC Cardiovasc. Imaging.

[5] Faletra F.F., Nucifora G., Ho S.Y. (2011). Imaging the atrial septum using real-time three-dimensional transesophageal echocardiography: Technical tips, normal anatomy, and its role in transseptal puncture. J. Am. Soc. Echocardiogr. Off. Publ. Am. Soc. Echocardiogr..

[6] Altiok E., Becker M., Hamada S., Grabskaya E., Reith S., Marx N., Hoffmann R. (2010). Real-time 3D tee allows optimized guidance of percutaneous edge-to-edge repair of the mitral valve. JACC Cardiovasc. Imaging.

[7] Faletra F.F., Pedrazzini G., Pasotti E., Petrova I., Drasutiene A., Dequarti M.C., Muzzarelli S., Moccetti T. (2013). Role of real-time three dimensional transoesophageal echocardiography as guidance imaging modality during catheter based edge-to-edge mitral valve repair. Heart.

[8] Faletra F.F., Pedrazzini G., Pasotti E., Muzzarelli S., Dequarti M.C., Murzilli R., Schlossbauer S.A., Slater I.P., Moccetti T. (2014). 3D TEE during catheter-based interventions. JACC Cardiovasc. Imaging.

[9] McIlwain E.F., Coon P.D., Einstein A.J., Mitchell C.K., Natello G.W., Palma R.A., Park M.M., Ranallo F., Roberts M.L. (2014). Radiation safety for the cardiac sonographer: Recommendations of the radiation safety writing group for the council on cardiovascular sonography of the american society of echocardiography. J. Am. Soc. Echocardiogr. Off. Publ. Am. Soc. Echocardiogr..

[10] Goldstein J.A., Balter S., Cowley M., Hodgson J., Klein L.W. (2004). Occupational hazards of interventional cardiologists: Prevalence of orthopedic health problems in contemporary practice. Catheter. Cardiovasc. Interv. Off. J. Soc. Card. Angiogr. Interv..

[11] Ross A.M., Segal J., Borenstein D., Jenkins E., Cho S. (1997). Prevalence of spinal disc disease among interventional cardiologists. Am. J. Cardiol..

[12] Wang S., Housden J., Singh D., Althoefer K., Rhode K. (2016). Design, testing and modelling of a novel robotic system for trans-oesophageal ultrasound. Int. J. Med. Robot. Comput. Assist. Surg..

[13] Ott L., Nageotte F., Zanne P., Mathelin M.D. (2011). Robotic assistance to flexible endoscopy by physiological-motion tracking. IEEE Trans. Robot..

[14] Abolmaesumi P., Salcudean S.E., Wen-Hong Z., Sirouspour M.R., DiMaio S.P. (2002). Image-guided control of a robot for medical ultrasound. IEEE Trans. Robot. Autom..

[15] Loschak P.M., Brattain L.J., Howe R.D. (2014). Algorithms for automated pointing of cardiac imaging catheters. International Workshop on Computer-Assisted and Robotic Endoscopy.

[16] Wang S., Housden J., Singh D., Rhode K. (2017). Automatic adjustments of a trans-oesophageal ultrasound robot for monitoring intra-operative catheters. IOP Conference Series: Materials Science and Engineering.

[17] Wang S., Singh D., Johnson D., Althoefer K., Rhode K., Housden R.J. (2016). Robotic ultrasound: View planning, tracking, and automatic acquisition of transesophageal echocardiography. IEEE Robot. Autom. Mag..

[18] Gao G., Penney G., Ma Y., Gogin N., Cathier P., Arujuna A., Morton G., Caulfield D., Gill J., Aldo Rinaldi C. (2012). Registration of 3D trans-esophageal echocardiography to X-ray fluoroscopy using image-based probe tracking. Med. Image Anal..

[19] Hutchinson S., Hager G.D., Corke P.I. (1996). A tutorial on visual servo control. IEEE Trans. Robot. Autom..

[20] Wang S., Singh D., Lau D., Reddy K., Althoefer K., Rhode K., Housden R.J. (2016). Probe tracking and its application in automatic acquisition using a trans-esophageal ultrasound robot. International Workshop on Computer-Assisted and Robotic Endoscopy.

[21] Huang J., Triedman J.K., Vasilyev N.V., Suematsu Y., Cleveland R.O., Dupont P.E. (2007). Imaging artifacts of medical instruments in ultrasound-guided interventions. J. Ultrasound Med..

[22] Stoll J., Ren H., Dupont P.E. (2012). Passive markers for tracking surgical instruments in real-time 3-D ultrasound imaging. IEEE Trans. Med. Imaging.

[23] Wu X., Housden J., Varma N., Ma Y., Rueckert D., Rhode K. (2013). Catheter tracking in 3D echocardiographic sequences based on tracking in 2D X-ray sequences for cardiac catheterization interventions. Proceedings of the 2013 IEEE 10th International Symposium on Biomedical Imaging (ISBI).

[24] Wu X., Housden J., Ma Y., Razavi B., Rhode K., Rueckert D. (2015). Fast catheter segmentation from echocardiographic sequences based on segmentation from corresponding X-ray fluoroscopy for cardiac catheterization interventions. IEEE Trans. Med. Imaging.

[25] Moore J.T., Wiles A.D., Wedlake C., Bainbridge D., Kiaii B., Trejos A.L., Patel R., Peters T.M. Integration of trans-esophageal echocardiography with magnetic tracking technology for cardiac interventions. Proceedings of the Medical Imaging 2010: Visualization, Image-Guided Procedures, and Modeling.

[26] Condino S., Calabrò E., Alberti A., Parrini S., Cioni R., Berchiolli R., Gesi M., Ferrari V., Ferrari M. (2014). Simultaneous tracking of catheters and guidewires: Comparison to standard fluoroscopic guidance for arterial cannulation. Eur. J. Vasc. Endovasc. Surg..

